# Consistent position of the superficial inferior epigastric vein (SIEV) during deep inferior epigastric perforator (DIEP) flap raise: A radiological analysis

**DOI:** 10.1016/j.jpra.2025.10.004

**Published:** 2025-10-17

**Authors:** Henry M Shepherd, Olivia L Sharp, Yousif F Yousif, Kieran T Power, Theo G Nanidis

**Affiliations:** Department of Plastic Surgery, The Royal Marsden Hospital, London, UK

**Keywords:** DIEP flap, SIEV, Microsurgery, Venous anatomy, Radiological analysis

## Abstract

The superficial inferior epigastric vein (SIEV) is often harvested and preserved with a deep inferior epigastric perforator (DIEP) flap for potential anastomosis in cases where venous congestion is anticipated or develops intraoperatively. There is often uncertainty regarding the exact location of the SIEV within the inferior incision of the DIEP flap. To our knowledge, no study has examined anatomical patterns of the vein at this level. This study was performed to determine the variability in location of the SIEV at the level of the inferior incision of the DIEP flap. We reviewed the preoperative CT angiograms of the last 50 sequential DIEP patients (100 hemiabdomens) to assess the distance of the SIEV from the midline at the level of the lower abdominal incision. The SIEV was absent or not clear radiologically at this level in one patient bilaterally and nine patients unilaterally. Of the 89 recorded measurements, the mean distance of the SIEV from the midline was 66 mm (range 50–83, SD 7). All measurements fell within 17 mm of the mean. We demonstrated that the position of the SIEV is consistent at the level of the inferior incision of a DIEP flap, and that this mean and range from the midline can be used to guide surgeons when attempting to identify this vessel.

## Background

The superficial inferior epigastric vein (SIEV) is an important anatomical structure when raising a deep inferior epigastric perforator (DIEP) flap, as it provides a key route of drainage for the superficial venous system of the abdominal wall.^1^ It is formed within the subcutaneous tissues and lies superficial to Scarpa’s fascia. It is a single vessel 82 % of the time and drains to the femoral vein in 95 % of patients.^1^^,^^2^

The SIEV is often harvested during a DIEP flap raise as an additional vein to augment outflow in cases where venous congestion is a problem.^3^ Once harvested, it can be anastomosed to another vein within the flap or to an external recipient vein. Exploration and identification of the SIEV occurs through the inferior DIEP incision. We observed that the location of the SIEV was confined to a narrow and reproducible margin either side of the midline, regardless of body habitus.

We performed this study to determine the variability in location of the SIEV at the level of the inferior incision of the DIEP flap. To the best of our knowledge, no previous study has assessed the anatomical position of the superficial inferior epigastric vein (SIEV) using preoperative CT imaging in a structured manner.

## Methods

We reviewed the preoperative CT angiograms of the last 50 sequential DIEP patients at our institution to assess the distance of the SIEV from the midline at the level of the lower abdominal incision. Patients who had an alternative flap reconstruction were excluded.

The level of the lower incision varies between patients and surgeons, however it typically falls between the anterior superior iliac spine (ASIS) and the pubic symphysis. For this study, we used a point halfway between these two radiological landmarks. Measurements were recorded from the midline to the SIEV on each hemi-abdomen at this level. In our institution, this level correlates well clinically with the point of harvest of the SIEV. If it was seen to divide, measurements of the lateral branch were taken.

Our CT angiography protocol is as follows; an 18G/20 G cannula is used and patients breath-hold to aid visualization of the venous network. Intravenous contrast is injected at 5 ml/s followed by a 50 ml saline flush. Scan acquisition is complete when there is full opacification of the femoral arteries. Images are compiled into maximum intensity projections (MIPs).

## Results

Of the 50 CT angiogram scans reviewed, the mean age was 54 years (range 34–47) and mean BMI was 26.5 (range 19.5–35.8).

At the measured level, the SIEV was absent or not clear radiologically in one patient bilaterally and nine patients unilaterally (one right, eight left). The total number of measurements recorded at this point were 89 (48 right, 41 left).

The mean distance of the SIEV from the midline at this point was 66 mm (range 50–83, SD 7, mean right 66 and mean left 67). All measurements fell within 17 mm of the mean. Six patients were documented as having lower abdominal scars such as from caesarean sections. 11/12 of these hemiabdomens had SIEVs visible on CTA.

## Discussion

Challenges with the venous drainage of a DIEP flap are more likely than issues with arterial inflow.^3^ The consequences of venous congestion can be devastating, with the potential for partial or complete flap failure. In these instances, the SIEV offers a lifeline as it can provide additional venous drainage.

The SIEV is harvested through the inferior incision of the DIEP flap. To our knowledge, this is the first study to examine the position of the SIEV at this level and to demonstrate its consistent position, regardless of body habitus. We found that the average distance from the midline was 6.6 cm. This was similar between right and left sides and for patients in a BMI range of 19.5–35.8. All vessels were found within a narrow (approximately) 3 cm range (5–8 cm from the midline). Using this range as a guide, surgeons can proceed with pace and efficiency outside of these measurements with the knowledge that inadvertent damage to the vein is less likely.

In eleven hemiabdomens, the superficial inferior epigastric vein (SIEV) could not be visualized. This may reflect limitations in image quality, or anatomical variables such as diminutive venous caliber. A marked asymmetry was observed in the distribution of non-visualization, occurring in eight left-sided and only one right-sided case. This is considered an incidental finding.

**Figure 1:**
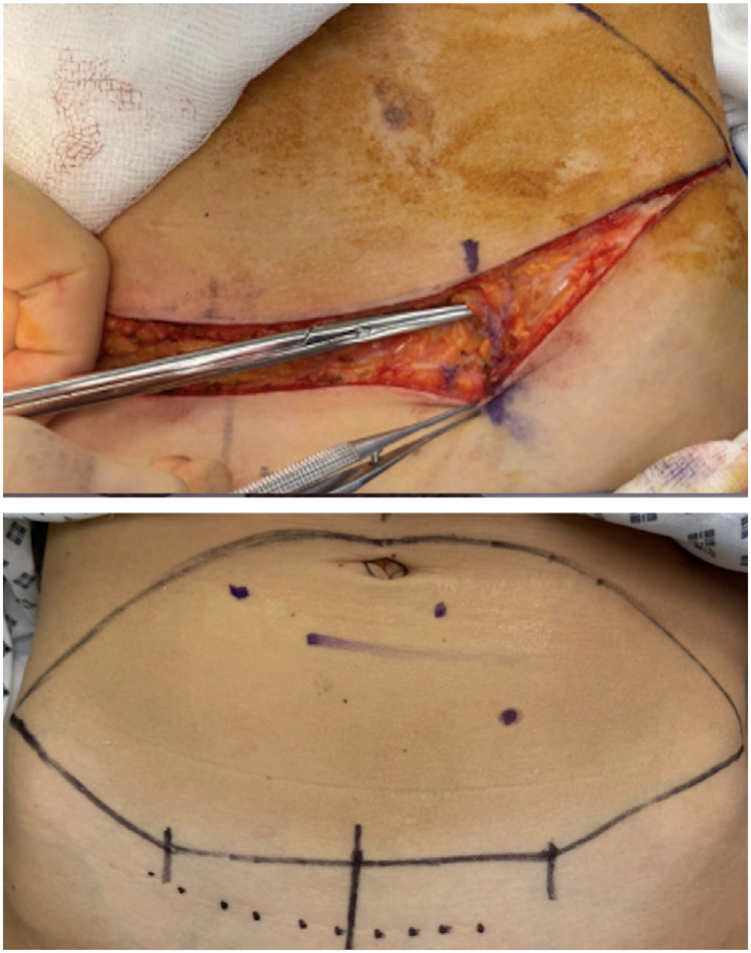
(a) Preoperative DIEP markings identifying 7 cm from either side of the midline on the lower incision and (b) an intraoperative image demonstrating the position of the SIEV at this marking.

**Figure 2:**
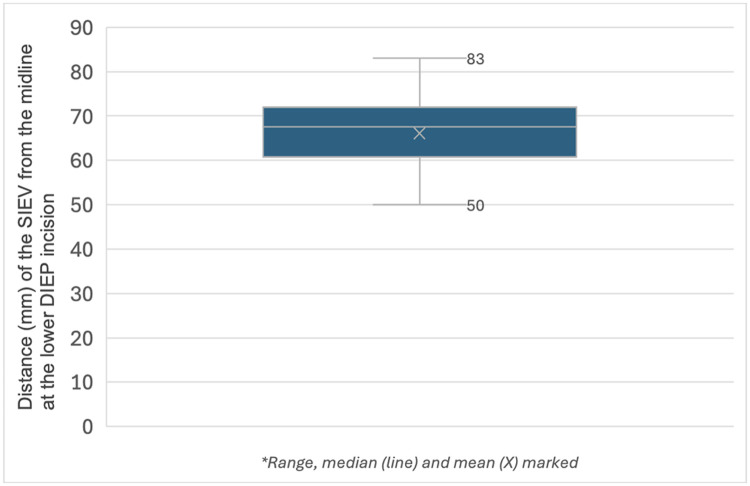
Distance (mm) of the SIEV from the midline at the approximate level of the lower DIEP incision.

In our institution, we base our lower DIEP incision around this point. We measure 7 cm each side of the midline to identify the approximate site of the SIEV and then mark a transverse line between these to form the central lower portion of the incision (Figure 1, Figure 2). The markings then taper superolaterally on each side as per standard abdominoplasty/DIEP design.

The main limitation of this study is correlating the location of the inferior incision to a point on an CT scan. Although the chosen point provides a standardized measurement between two fixed structures that approximates the level of the inferior incision, the actual level of the incision will differ between patients and surgeons.

## Conclusion

We have demonstrated that the position of the SIEV is consistent at the level of the inferior incision of a DIEP flap, and that this mean and range can be used to guide surgeons when identifying this vessel.

## Funding

The authors have no financial interest in declaring the content of this article. No funding was received for this article.

## Author contributions statement

All authors contributed to the final version of this manuscript.

## Ethical statement

This study involved the retrospective analysis of anonymized radiological data. In accordance with applicable guidelines and regulations, formal ethical approval was not required. All procedures complied with the principles of the Declaration of Helsinki.

## Declaration of competing interest

The authors declare no potential conflict of interest.
